# Developing a patient-centered community-based model for management of multi-drug resistant tuberculosis in Uganda: a discrete choice experiment

**DOI:** 10.1186/s12913-021-07365-5

**Published:** 2022-02-05

**Authors:** Rita Makabayi-Mugabe, Joseph Musaazi, Stella Zawedde-Muyanja, Enock Kizito, Hellen Namwanje, Philip Aleu, Danielle Charlet, Debora B. Freitas Lopez, Haley Brightman, Stavia Turyahabwe, Abel Nkolo

**Affiliations:** 1 The Infectious Diseases Institute, College of Health Sciences, Makerere University, P.0.Box 22418, Kampala, Uganda; 2 USAID-Defeat TB Project, University Research Co. LLC, Plot 40 Ntinda II Road, P.O. BOX 28745, Kampala, Uganda; 3 University Research Co. LLC, Chevy Chase, 5404 Wisconsin Avenue, Suite 800, Maryland 20815 USA; 4 Ministry of Health-National TB and Leprosy Division, Kampala, Uganda

**Keywords:** Community-based care, Directly observed therapy, Multi-drug resistant tuberculosis, Uganda, Discrete choice experiment

## Abstract

**Background:**

The advent of all-oral regimens for the management of multi-drug resistant tuberculosis (MDR-TB) makes the implementation of community-based directly observed therapy (CB-DOT) a possibility for this group of patients. We set out to determine patient preferences for different attributes of a community-based model for the management of MDR-TB in Uganda.

**Methods:**

The study was conducted at five tertiary referral hospitals. We used a parallel convergent mixed methods study design. To collect quantitative data, we conducted a discrete choice experiment (DCE) with three different attributes of community-based care (DOT provider, location of care, and type of support) combined into eight choice sets, each with two options and an opt-out. We elicited patient reasons for selection of each choice set using qualitative methods. We fitted a mixed logit choice model to determine patient preferences for different attributes of community-based care and estimated the relative importance of each attribute using the range method. and used deductive thematic analysis to understand the reasons for the choices made.

**Results:**

From December 2019 to January 2020, we interviewed 103 patients with MDR-TB. We found that all the three attributes considered were important predicators of choice. The relative importance of each attribute was as follows; the type of additional support (relative importance 36.2%), the location of treatment delivery (33.5%), and the type of DOT provider (30.3%). Participants significantly valued treatment delivered by community health workers (CHWs) or expert clients over that delivered by a family member, treatment delivered at home over that delivered at the workplace, and monthly travel vouchers as the form of additional support over phone call or SMS reminders. Subgroup analyses showed significant differences in preference across HIV status, age groups and duration on MDR-TB treatment, but not across gender.

The preferred model consisted of a CHW giving DOT at home and travel vouchers to enable attendance of monthly clinic follow-up visits to tertiary referral hospitals for treatment monitoring. Qualitative interviews revealed that patients perceived CHWs as knowledgeable and able to offer psychosocial support. Patients also preferred to take medication at home to save both time and money and lower the risk of facing TB stigma.

**Conclusion:**

People with MDR-TB prefer to be supported to take their medicine at home by a member of their community. The effectiveness of this model of care is being further evaluated.

**Supplementary Information:**

The online version contains supplementary material available at 10.1186/s12913-021-07365-5.

## Background

Multi-drug resistant tuberculosis (MDR-TB), defined as resistance to both isoniazid and rifampicin, the two major first line anti-TB medicines, threatens global TB control efforts and remains a major public health concern in many countries [1]. Globally, case detection and treatment success rates for MDR-TB are suboptimal. In Uganda, only 64% of those started on treatment for multi-drug resistant TB in 2016 were successfully treated while an estimated 19% died and 15% were lost to follow up [2]. These suboptimal treatment outcomes are a potential risk for the development and spread of further resistance to TB treatment [3].

Uganda currently implements a mixed model of care for MDR-TB characterized by initial hospitalization for two to eight weeks followed by ambulatory directly observed therapy at a public or private health facility near the patient’s home [4]. However, delivery of care through health facility-based directly observed therapy (HF-DOT) has been documented to significantly contribute to poor treatment outcomes particularly in resource limited settings [5]. Patients who receive care through HF-DOT experience various inconveniences (e.g., travel and waiting times) and incur significant direct (e.g., transport costs) and indirect costs (e.g., time lost from work) that hinder successful treatment completion [6, 7]. In the management of both drug susceptible and drug resistant TB, community-based treatment support models have been associated with improved treatment outcomes and increased cost effectiveness compared to health facility-based models [8–11].

Since March 2018, a shorter treatment regimen has been the standard of care for patients with MDR-TB without extrapulmonary TB or resistance to fluoroquinolones or injectable drugs. This regimen consists of six drugs; kanamycin (Km), moxifloxacin (Mfx), Ethionamide (Eto), clofazimine (Cfz), pyrazinamide (Z), high dose Isoniazid (H) and ethambutol (E) given in two phases; an intensive four-to-six-month period and a five-month continuation phase [4]. This treatment regimen is better tolerated, and in clinical trials resulted in better treatment outcomes than the two-year treatment regimen [12]. However, this regimen also has lower tolerance for lapses in adherence [4, 12, 13]. The provision of effective adherence support for patients receiving the shorter MDR-TB regimen is therefore a priority intervention for the National TB and Leprosy Program (NTLP). The provision of community-based adherence support has the potential to decrease patient costs [9, 11] and subsequently improve patient retention and lead to better treatment outcomes [3]. Further, the use of community health workers (CHWs) to offer treatment adherence support, particularly in the continuation phase of treatment, is recommended in the Uganda national guidelines for programmatic management of drug resistant Tuberculosis [4] but has not yet been implemented [14]. In order to design a patient centered community-based model for the management of multi-drug resistant TB, we set out to assess patient preferences for different attributes of community-based care.

## Methods

### Study setting

The study was carried out at five tertiary referral hospitals purposively selected because they provide care to about 80% of patients started on treatment for multi drug resistant TB in Uganda every year. At the time of the study, 302 MDR-TB patients were enrolled into care across study sites.

### Study design

We used a parallel convergent mixed methods study design to collect both quantitative and qualitative data on patient preferences for different attributes of community-based care.

### Sample size

In our discrete choice experiment, the number of tasks (t) was 8, the number of alternatives per task - not including the null alternative (a) was 2, and the number of levels for each attribute (c) was 3. Using the Orme and Johnson sample size calculation formula for discrete choice experiment [15]: *N* > 500c/(t × a) [16] and adding 10% for non-response, our estimated sample size was 104 respondents.

### Selection of respondents

Between December 2018 and August 2019, 490 patients were started on treatment for MDR-TB in Uganda. Of these 302 patients were on the shorter, all oral treatment regimen and were therefore eligible for the study. We included patients 18 years or older who had completed the intensive phase of treatment and excluded 20 patients who had missing contact information. We used sampling proportionate to size to determine the number of patients to be selected from each hospital, and simple random sampling to select the patients to be interviewed at each hospital.

### Data collection

We collected quantitative data on patient preferences using a discrete choice experiment (DCE) [17]. The DCE reveals how individuals’ value selected attributes of a program or service and allows for estimation of the relative importance of different aspects of care, the trade-offs between these aspects, and the total satisfaction respondents derive from health care services [18]. We chose three attributes (DOT provider, location of service, and provision of additional support) each with two or three attribute levels (Table 1). Attributes used in this study were chosen from an initial exploratory study carried out six months prior.

**Table 1:** DCE attributes and levels

Attribute	Level	Attribute definitions for this study
**DOT Provider**	Expert client	An individual who was treated for MDR-TB and recovered with good treatment outcomes. This person serves as an example in their community and helps others overcome a similar condition.
	Family member	An adult older than 18 years living with the MDR-TB patient in the same household.
	Community health worker	A person with basic health related training, who lives not more than an hour’s walk away from the patient’s home
**Location**	Home	A place where one lives permanently, especially as a member of a family.
	Work	A place of employment to earn a living. It may be formal, informal, self-employed, or having an employer. In this study, this also included school for students.
**Support type**	SMS reminders	An appointment text message sent to a mobile telephone one week prior to the patients follow up clinic visit.
	Call reminders	A telephone call made one week prior to the patients follow up clinic visit at the tertiary hospital for treatment monitoring
	Travel vouchers	A travel ticket worth 13$ issued to the patient by the tertiary hospital with a date on which to return to the tertiary hospital for treatment monitoring.

#### Attributes and levels

To identify the model attributes, the research team reviewed available policy documents and guidelines on provision of DOT for TB as well as published literature on community based models of care delivery [4, 19, 20]. Using a semi structured questionnaire, a pilot study was carried out at an urban hospital among patients with drug resistant TB to elicit their views on the proposed attributes and attribute levels. Results from this pilot study informed the final selection of attributes for this study e.g., healthcare workers were dropped from choice of DOT provider due to perceived unavailability by the respondents.

Using a fractional factorial design, we chose eight choice sets. Recommendations in the literature [21, 22] have shown that more than eight choice tasks are a cognitive and time burden for participants. We included an opt-out response category so that respondents could choose “neither” choice set to reflect dissatisfaction with either potential CB-DOT model. The final design had 96% d-efficiency (Table 2). To cater for limited literacy levels among the respondents, visual representations of each choice set were developed and used during data collection (Additional file 1). Additional data on age, gender, marital status, date of treatment initiation, duration on MDR-TB treatment, and underlying co-morbidities was abstracted from health facility records. Data was collected by research assistants with experience collecting quantitative data who were trained on the study tools and supervised by the research team. Data was then entered into an electronic web-based management system called TB management information system (tbmis).

**Table 2:** DCE choice sets each with an opt out option

Choice Set	Choice set alternatives	Attributes		
		Location of care	DOT provider	Support type
1	I	work	CHW	Travel vouchers
	II	Home	Expert Client	Call reminders
	III	Neither		
2	I	Home	Family member	SMS reminders
	II	work	Expert Client	Travel vouchers
	III	Neither		
3	I	Home	Family member	SMS reminders
	II	work	CHW	Call reminders
	III	Neither		
4	I	Home	Expert Client	SMS reminders
	II	Home	Family member	Call reminders
	III	Neither		
5	I	Home	Family member	Travel vouchers
	II	Home	CHW	SMS reminders
	III	Neither		
6	I	Work	CHW	SMS reminders
	II	Home	Expert Client	Travel vouchers
	III	Neither		
7	I	Work	Expert Client	SMS reminders
	II	Home	CHW	Call reminders
	III	Neither		
8	I	Work	Expert Client	Call reminders
	II	Home	CHW	Travel vouchers
	III	Neither		

8 choice sets. D-efficiency after iteration 0.9607

Qualitative data was collected through patient interviews carried out after each choice set was presented to all study participants to find out the reasons for choices made for each set of attributes. Face to face interviews were held in local dialects to ascertain reasons for choosing each choice set. The interviews were carried out within the hospital premises and lasted from 10 to 30 min. The interview scripts were transcribed and entered into NVivo software version 12 for analysis.

### Data analysis

#### Quantitative data

Quantitative analyses were conducted using STATA software version 15.1 (StataCorp, College Station, Texas, USA). We described participants’ characteristics using descriptive summaries; frequency and percentages for categorical variables, median and inter-quartile range for count data like age, and duration on MDR-TB treatment.

#### DCE analysis to determine the patients’ preferred MDR-TB CB-DOT model

We used a mixed logit (MIXL) model (with 1000 Halton simulation draws and normal distribution) to determine the preferred attributes for community-based care for MDR-TB treatment, because MIXL estimates both the participants’ degree of preference and degree of heterogeneity in preference across study participants [21, 22]. Conditional relative importance of the attributes was estimated using range method [21, 22].

Subgroup analyses were conducted to examine whether participants’ preferences differ by age [< 35, ≥35 years], gender [female, male], HIV status [negative, positive], and duration on MDR-TB treatment [< 6, ≥6 months]. The significance level for all the analyses was determined at 5%.

To determine the preferred model of care, we calculated utility scores by substituting coefficients (preference weights) into the model equation (with different combination of attributes levels). The attributes combination with highest total utility score was considered as the preferred model.

#### Qualitative data

A deductive approach with descriptive thematic coding was used to analyse data using NVivo Version 12. The coding framework was developed using open coding by an experienced behavioural scientist (AT) after reading 5% of all transcripts. It was later reviewed by two members of the research team (RMM and DC). Subsequent analyses of transcripts were carried out by two members of the research team (RMM and DC) who then compared and discussed their findings. Discrepancies were resolved by mutual agreement. To ensure trustworthiness, transcripts were coded independently, compared, discussed [18]. Interview transcripts were reviewed for content related to the research question. Codes were compared and similar codes aggregated, and consensus sought for validation coding. There was flexibility to accommodate emergent new themes as coding evolved. Data within and across themes were synthesized to generate an understanding of why certain attribute choices were preferred.

## Results

### Socio-demographics

From December 2019 to January 2020, 103 participants were interviewed. The majority, 58.3% were male, HIV negative (61.2%), and earned less than 1$/day (65.1%). The median age was 37 years (inter-quartile range [IQR] 30 to 47 year) (Table 3).

**Table 3:** Baseline Characteristics of Study Participants

Characteristics	Number (%), ***N*** = 103
**Gender**	
Female	43 (41.7)
Male	60 (58.3)
**Age in years**, median (IQR)	37 (30–47)
**Age categories**	
20–34	42 (40.8)
35–80	61 (59.2)
**Marital status**	
Single/Single/Divorced/Separated	48 (46.6)
Married	55 (53.4)
**Occupation**	
Farmer	37 (35.9)
Business	24 (23.3)
Employed	33 (32.1)
Unemployed	9 (8.7)
**Daily income** ($)^a^	
< 1$	67 (65.1)
≥1$	36 (34.9)
**Education**	
None	11 (10.8)
Primary	58 (56.9)
Secondary	24 (23.5)
Tertiary	9 (8.8)
**HIV status**	
Negative	63 (61.2)
Positive	40 (38.8)
**Duration on MDR-TB treatment** (months)^a^	
Median (IQR)	10 (5–16)
Categories	
< 6 months	31 (30.4)
≥ 6 months	71 (69.6)

^a^Missing values; Educational level (1), Duration on MDR-TB treatment (1)

### Discrete choice model analysis

#### Preference for specific attributes of community-based care

Results for the mixed logit model (MIXL model) are presented in Table 4. Significant differences were observed between levels of each attribute, implying that all three attributes considered were important predicators of choice. The relative importance of each attribute was as follows; type of additional support (relative importance 36.2%), location of treatment delivery (33.5%), and type of DOT provider (30.3%). Participants significantly valued: (1) treatment delivered by CHWs or expert clients over that delivered by a family member; (2) treatment delivered at home over that delivered at the workplace; and (3) monthly travel vouchers as the form of additional support over phone call or SMS reminders.

**Table 4:** Results of random parameter logit model (mixed logit model) for the MDR-TB CB-DOT model attributes

Attributes and levels^a^	Coefficients	95%CI	***P***-value	SD	95%CI	***P*** value^**†**^	Relative importance
**Constant**	0.52	0.30–0.74	< 0. 01	-	-	-	-
**DOT provider**							**30.3%**
Family (reference)							
Community health work	1.13	0.68–1.59	< 0. 01	1.85	1.33–2.37	< 0.01	
Expert client	0.89	0.46–1.32	< 0. 01	1.77	1.24–2.30	< 0.01	
**Location**							**33.5%**
Workplace (reference)							
Home	1.25	0.91–1.59	< 0. 01	1.19	0.80–1.57	< 0.01	
**Support**							**36.2%**
SMS (reference)							
Phone call reminders	0.70	0.41–0.99	< 0. 01	0.19	0.95–1.33	0.74	
Travel vouchers	1.35	1.04–1.65	< 0. 01	0.55	0.04–1.05	0.033	

^a^Dummy coded attributes (coefficient of the reference category is constrained to be 0)

*CI* Confidence interval, *SD* standard deviation for preference heterogeneity (random component of the model coefficients)

† *P* value testing the hypothesis that standard deviation (heterogeneity across individuals’ preferences) equals ‘0’

Number of observations = 2472

The standard deviation estimates, indicating the preference variation among participants per attribute and level, were all significantly different from “0” (*P* values> 0.01) except for the phone call reminders (P value 0.74). The highest variation was on preference of treatment delivered by community health worker as DOT provider.

### Sub-group analysis

Subgroup analyses showed significant difference for at least one attribute across HIV status, age groups and duration on MDR-TB treatment. There was no significant difference for preference of attributes across gender. Results for the three significant subgroup analyses are presented in Tables 5, 6 and 7.

**Table 5:** Results of random parameter logit model subgroup analysis for HIV status of respondent

Attributes and levels^a^	HIV Negative			HIV Positive			***P*** value†
	Coefficient	SD	Relative importance	Coefficient	SD	Relative importance	
**DOT provider**			**34.2**			**24.2**	
Family (reference)	–	–		–	–		
Community health work	1.22	1.78		0.95	2.07		0.356
Expert client	0.89	1.50		0.84	2.37		0.741
**Location**			**27.5**			**43.0**	
Workplace (reference)							
Home	0.98	1.11		1.69	1.26		0.042
**Support**			**38.4**			**32.8**	
SMS (reference)							
Phone call reminders	0.76	0.56		0.56	0.01		0.668
Travel vouchers	1.37	0.47		1.29	0.53		0.582

^a^Dummy coded attributes (coefficient of the reference category is constrained to be 0)

*CI* Confidence interval, *SD* standard deviation for preference heterogeneity (random component of the model coefficients)

Number of observations = 1512 (HIV negative), 960 (HIV positive)

† *P* value for indicating statistical difference in preference weights of attribute levels by HIV status

**Table 6:** Results of random parameter logit model subgroup analysis for age groups of respondents

Attributes and levels^a^	Age 20–34 years			Age ≥ 35 years			***P*** value
	Coefficient	SD	Relative importance	Coefficient	SD	Relative importance	
**DOT provider**			**28.4**			**58.6**	
Family (reference)	–	–		–	–		
Community health work	0.80	2.25		1.37	1.57		0.016
Expert client	1.13	2.18		0.79	1.60		0.888
**Location**			**40.7**			**33.7**	
Workplace (reference)							
Home	1.62	1.65		1.09	0.92		0.495
**Support**			**30.9**			**7.7**	
SMS (reference)							
Phone call reminders	0.87	0.58		0.62	0.06		0.743
Travel vouchers	1.23	0.76		1.43	0.21		0.169

^a^Dummy coded attributes (coefficient of the reference category is constrained to be 0)

*CI* Confidence interval, *SD* standard deviation for preference heterogeneity (random component of the model coefficients)

Number of observations = 1008 (age 20–34 years), 1464 (age ≥ 35 years)

**Table 7:** Results of random parameter logit model subgroup analysis for duration of MDR-TB treatment

Attributes and levels^a^	On treatment < 6 months			On treatment ≥ 6 months			***P*** value
	Coefficient	SD	Relative importance	Coefficient	SD	Relative importance	
**DOT provider**			**16.7**			**36.7**	
Family (reference)	–	–		–	–		
Community health work	0.58	1.94		1.49	1.77		0.018
Expert client	0.32	1.79		1.16	1.87		0.017
**Location**			**48.0**			**27.8**	
Workplace (reference)							
Home	1.67	1.27		1.13	1.16		0.169
**Support**			**35.3**			**35.5**	
SMS (reference)							
Phone call reminders	0.73	0.99		0.66	0.11		0.546
Travel vouchers	1.23	0.33		1.44	0.62		0.543

^a^Dummy coded attributes (coefficient of the reference category is constrained to be 0)

Coefficients – indicates preference weights of attribute levels.

*CI* Confidence interval, *SD* standard deviation for preference heterogeneity (random component of the model coefficients)

Number of observations = 744 (on treatment< 6 months), 1704 (on treatment ≥6 months). Total observation <overall (2472) because 1 participant had missing duration on treatment

The attribute valued highest by HIV negative MDR-TB patients valued was the additional treatment support (relevant importance of 38.4%), while that valued highest by HIV positive MDR-TB patients was the treatment location (43%). The subgroup analysis by age groups and duration on MDR-TB treatment, indicated that young patients (below 35 years) and those who had been on MDR-TB treatment for < 6 months placed the highest value on the treatment location whereas older persons and those who had been on treatment for ≥6 months placed the highest valued on the DOT provider.

### Preferred hypothetical models (combination of attributes) of community-based care

Substituting the preference weights (coefficients) into the MIXL model to calculate total utility scores for a combination of attributes, the preferred community-based care model (i.e., set of attributes profile with highest total utility score) was a CHW giving treatment at the patient’s home and the patient receiving monthly travel vouchers for additional support. The models of care with the highest preference weights contained travel vouchers or treatment delivered at home (Table 8).

**Table 8:** Hypothetical MDR-TB CB DOT models ranks and utility scores

Profile	Utility score	Rank
H2 CHW/Treatment delivered at home/Travel voucher	4.25	1
F2 Expert clients/Treatment delivered at home/Travel voucher	4.01	2
G2 CHW/Treatment delivered at home/Phone call reminder	3.61	3
A2 Expert client/Treatment delivered at home/Phone call reminder	3.36	4
E1 Family member/Treatment delivered at home/Travel voucher	3.12	5
A1 CHW/Treatment delivered at work/Monthly travel voucher for monthly appointments	3.00	6
E2 CHW/Treatment delivered at home/SMS reminder for monthly appointments	2.90	7
B2 Expert client/Treatment delivered at work/Monthly travel voucher for monthly appointments	2.76	8
D1 Expert client/Treatment delivered at home/SMS reminder for monthly appointments	2.66	9
D2 Family member/Treatment delivered at home/Phone call reminder for monthly appointments	2.48	10
C2 CHW/Treatment delivered at work/Phone call reminder for monthly appointments	2.36	11
H1 Expert client/Treatment delivered at work/Phone call reminder for monthly appointments	2.11	12
C1 Family member/Treatment delivered at home/SMS reminder for monthly appointments	1.77	13
B1 Family member/Treatment delivered at home/SMS reminders for monthly appointments	1.77	13
C1 Family member/Treatment delivered at home/SMS reminder for monthly appointments	1.66	15
F1 CHW/Treatment delivered at work/SMS reminder for monthly appointments	1.41	16

***Utility score*** *calculated by substituting estimates of preference weights of attributes into the derived utility model*

***Utility score (V)*** *= .5230616 + 1.132371 (c_chw) + .8881919 (c_expert) + 1.250887 (l_home) + .7014401 (s_phcall) + 1.346391 (s_tvouch)*

### Qualitative results

The reasons for choice are presented around the attributes of DOT provider, location of care and type of support. Several key themes emerged from the data regarding the preferred attributes of community-based model of care and quotes documented to provide the patients perspective.

#### DOT provider

##### Theme: DOT provider type training and experience

Respondents reported that a CHW or expert client was preferred because they were trained and knowledgeable. In addition, they had the ability to offer adherence and psychosocial support including guidance on how to take their medications because of their professional or personal experience. Further, patients felt that expert clients could be more empathetic because they have been through similar experiences. They therefore trusted them to maintain confidentiality in the process of offering care.

*“A community health worker encourages you to take medicine than any other person. Also provides counselling to the patient and explains the benefit of taking medicines on time.”* **[20yr., Female, Single]**

*“I prefer an expert client to support me since he has been through the same situation. Expert client has the best experience ever [I] will be able to share with him.”* **[33yr., Male, Married]**

*“I prefer a CHW because they are knowledgeable, and I can trust them with my life.”* **[54yr., Male, Married]**

Family members were least preferred and viewed as unable to offer the support needed.

*“... family members, they sympathize so much and may discourage you from sticking to your treatment schedules as required and also they lack experience with this type of TB disease.”* **[22yr., Female, Single]**

#### Location of care

##### Theme: convenience and confidentiality

Patients preferred to receive care from home citing privacy and a lower risk of TB-related stigma. They also felt that receiving care at home saves them time and money and presents an opportunity for health education for their family.

*“The treatment at home also is a bonus, because the community cannot discriminate if confidentiality is kept by the expert client."* **[29yr., Female, Divorced/separated]**

*“Treatment at home and monthly travel vouchers for monthly appointments is my preferred choice because there is time management while taking drugs since no out movement.”*

**[66yr., Male, Married]**

*“It becomes less costly when I get treatment at home.”* **[31yr., Male, Married]**

The workplace was not preferred by the majority of the respondents. It was associated with stigma and fear for loss of employment or clients.

*“I feel confidentiality is not possible at my workplace where I could easily be seen and get stigmatized.”* **[22yr., Female, Divorced/separated]**

*“… if visited at work, I could easily suffer from the gossip by workmates when they eventually learn about my condition, this could easily make me lose clients.”* **[55yr., Female, Divorced/separated]**

#### Support type

##### Theme: travel vouchers address patient needs

Participants felt that monthly travel vouchers were the best way to promote appointment keeping as they provided relief from worrying about transport costs and money left over transport fares could be used to meet other needs like food, other household items, payment of debts, savings and farming amongst others.

“*Monthly travel vouchers are the best for me because I will be knowing that transport is readily available and even the ticket will be reminding me of the clinic appointment date.”* **[40yr., Male, Married]**

*“…because the money given for the travel voucher, the balance from it I can use it to buy other things and also do farming with it.”* **[52yr., Male, Married]**

##### Sub-theme: treatment support reminders

Some of the respondents did not prefer other types of patient support, e.g., SMS or phone calls, due to various limitations surrounding their use which included:

*“The message (SMS) reminders would work for me since am partially deaf and cannot easily hear through a phone call….*” **[43yr., Female, Divorced/separated*]***

*“….SMS reminders are not good for me because I don't know how to read but may be if they call me and give transport to the clinic.”* **[40yr., Male, Married]**

## Discussion

We carried out a parallel convergent mixed methods study to determine patient preferences for different attributes of community-based care. We found that people with MDR-TB preferred care to be provided at home by a CHW or an expert client who is a member of the community and as additional support, to receive travel vouchers to enable attendance at monthly clinic follow-up visits. CHWs and expert clients were viewed as knowledgeable, experienced, empathetic, and skilled to properly counsel and guide patients on how to manage side effects. They were also seen as able to maintain confidentiality in the process of offering care. Family members were viewed as lacking adequate MDR-TB related knowledge and patients were skeptical of their ability to offer the support required.

Preference for and patient satisfaction with lay providers has also been observed elsewhere, such as in rural Swaziland where DOT and administration of injectable forms of MDR-TB medication was delegated to trained community treatment supporters [23]. In that study, preference for lay providers was driven by their ability to offer adherence counselling, confidentiality, and perceived lower treatment costs, reasons similar to those given by patients in our study.

The majority of respondents preferred home care noting it provides privacy, safety, comfort, and an opportunity for health education including infection prevention and control at the family level. Further, patients viewed home-based care as being less costly as it saves on time spent accessing care and daily transportation costs to follow-up health facilities. The workplace was perceived as a possible source of stigma that could lead to loss of clientele for small business owners or loss of employment. Similar to our study, findings from rural northern Uganda [24] showed that home-based care was acceptable to both patients and health providers noting that it is safe, conducive to recovery and time saving. This study further showed that home-based care enabled psychosocial support. In our study, however, psychosocial support was mentioned as a reason for preference for a certain provider type rather than place of care.

In Bangladesh, a decentralized, community-based treatment program for patients with drug-resistant tuberculosis used home-based care DOT to address various needs of MDR-TB patients. It was a patient’s preferred approach evidenced by their retention in care resulting into improvement in other treatment outcomes [25]. Similarly, a quasi-experimental study done in India showed that home-based care was associated with low stigma [26] similar to our study findings. In rural South Africa, MDR-TB patients preferred to receive MDR-TB and HIV care at home, and this was associated with reduced levels of rejection creating strong emotional bonds between patients, families and communities that is critical to health [27]. The home is seen as a place conducive for recovery and offers both psychological and emotional support needed to enable healing [28]. In the sub-group analyses, more MDR-TB patients who were HIV co-infected preferred to be treated at home than those who were HIV negative. This may be related to the fact that in 2017, Uganda rolled out differentiated service delivery models for persons infected with HIV that included community-based drug delivery options [20]. It is possible that clients’ positive experience with these care delivery models positively influenced their choice for home-based care [29].

Despite documented evidence that digital mobile technologies are useful in supporting TB care [30], majority of participants preferred monthly travel vouchers over mobile-based support, such as SMS and phone call reminders. In our study, varying degrees of literacy and hearing loss due to drug toxicity probably decreased the preference for mobile technologies. In addition, the utility of the travel voucher in meeting other household needs besides travel to the monthly hospital appointment served as a main driver for this choice. The majority of our respondents earned only about a dollar a day and were receiving a transport voucher worth about 50 dollars a month from the NTLP under its ongoing “enabler program”. The travel voucher therefore shielded them against incurring catastrophic costs during their treatment. In Uganda, a recent study done to examine costs incurred during TB treatment showed that that more than half of households affected by TB experienced catastrophic costs, defined as spending more than 20% of their annual income on TB and these costs were 30 times higher among MDR-TB patients compared to drug sensitive tuberculosis (DS-TB) patients [31]. The major drivers of cost according to the Uganda TB cost survey were non-medical and included transportation, food, and nutritional supplements. Many respondents in our study reported that they could use money left over from the travel voucher to buy food. It is important to consider the unique needs of the sub-groups and the drivers of their behaviors so as to tailor interventions to address their specific needs. Further, due to the current COVID-19 pandemic, innovations that reduce the need for health facility visits while still providing additional support to meet client needs will become increasingly relevant [32].

The study had several strengths. We had regional representation across the country, involved health facilities that treat more than 80% of the MDR-TB cases. The results presented here are representative of patients receiving care for MDR TB across the country. In addition, we used patients who had been on treatment for MDR-TB. Their preferences were therefore grounded in their lived experiences with MDR-TB care. In addition, our study used pictorial questionnaires to aid understanding of choice sets and avoid strategic bias which could result in misrepresentation of preferences. However, our study had some limitations; we used routinely collected data which is prone to missing data. However, efforts were made to minimize this by training research assistants on quality data collection, triangulation of data sources and respective standard operating procedures prior to data collection. Finally we did not include children and other risk populations, like pregnant women so their preferences are not represented in our findings. Future studies could include children and other vulnerable populations so that their views are taken into consideration.

## Conclusions and recommendations

Our respondents preferred to take their medicines at home supported by a member of their community but revealed a critical need for additional support to help mitigate the costs of accessing care. These patient preferences should be incorporated into future MDR-TB treatment approaches designed by the National TB Program. The feasibility and effectiveness of these models of care should be further evaluated. Studies to determine the feasibility and effectiveness of our preferred patient care model are underway.

## Supplementary Information

**Additional file 1.** Pictorial to support DCE choices.

## Data Availability

The datasets used and/or analyzed during the current study available from the corresponding author on request.
