# Supplementary material for: Developing a patient-centered community-based model for management of multi-drug resistant tuberculosis in Uganda: a discrete choice experiment
Source: BMC Health Serv Res. 2022 Feb 5;22:154. doi: 10.1186/s12913-021-07365-5 (PMC8817775; doi:10.1186/s12913-021-07365-5)

Option 1

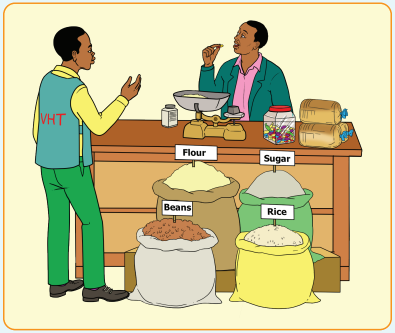

Community Health Worker giving treatment at work

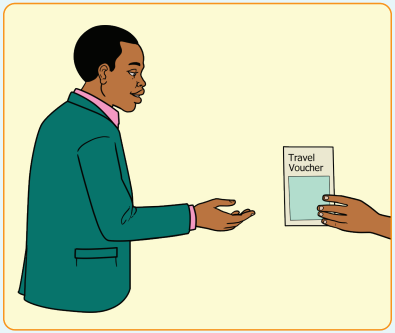

Monthly travel vouchers for monthly appointment

Option 2

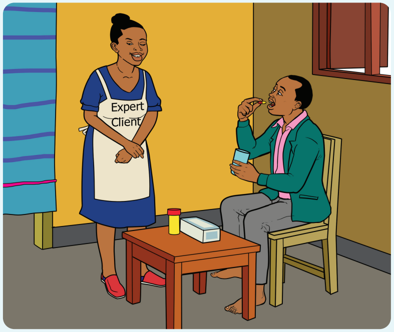

Expert Client giving treatment at home

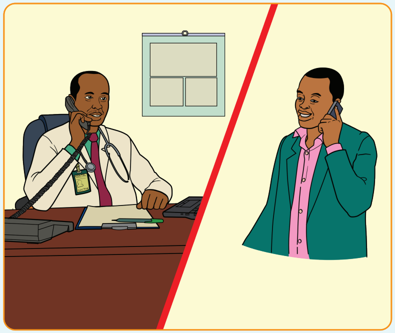

Phone call reminders for monthly appointments

Option 3

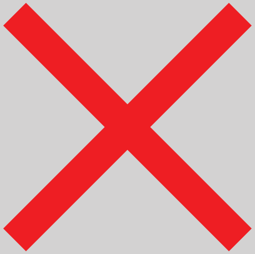

Supplement: Supplementary file 1 — Additional file 1. Pictorial to support DCE choices. [file 12913_2021_7365_MOESM1_ESM.pdf]
